# The HIV epidemic in Latin America: a time to reflect on the history of success and the challenges ahead

**DOI:** 10.1002/jia2.25468

**Published:** 2020-03-01

**Authors:** Brenda Crabtree‐Ramírez, Pablo F Belaunzarán‐Zamudio, Claudia P Cortes, Miguel Morales, Omar Sued, Juan Sierra‐Madero, Pedro Cahn, Anton Pozniak, Beatriz Grinsztejn

**Affiliations:** ^1^ Departamento de Infectología Instituto Nacional de Ciencias Médicas y Nutrición, Salvador Zubirán Tlalpan Mexico; ^2^ Fundación Arriarán University of Chile Santiago Chile; ^3^ Taller Venezolano de VIH Caracas Venezuela; ^4^ Fundación Huésped Investigaciones Clínicas Buenos Aires Argentina; ^5^ Chelsea and Westminster Hospital NHS Foundation Trust and Imperial College London London UK; ^6^ Instituto Nacional de Infectologia Evandro Chagas Fundacao Oswaldo Cruz Rio de Janeiro Brazil

**Keywords:** Latin America, HIV epidemic, migration, HIV, PrEP in Latin America, ART, HIV testing

According to Joint United Nations Programme on HIV/AIDS (UNAIDS) data, 1,900,000 adults and children were living with HIV in Latin America and the Caribbean in 2018 1, where overall prevalence was 0.5%. Latin America's HIV epidemic is concentrated among men who have sex with men, transgender women, sex workers and people who inject drugs 2. In comparison, the Caribbean has a smaller population of people living with HIV (PLHIV), but a generalized epidemic with an overall HIV prevalence of 1.2% and women accounting for half of all infections 3. Within a short time span and through extraordinary efforts, programmes for universal access to antiretroviral therapy (ART) were rolled out in all countries in the region 4 despite the absence of previous infrastructure for HIV care provision and the lack of international financial support for these programmes in most of the countries.

From 2003 to 2008, the number of people on ART doubled and steadily increased afterwards; by 2017, approximately 1.2 million PLHIV (61%) were receiving ART, lagging only after high‐income countries (78%) 1. Furthermore, mortality after ART initiation has decreased and is very similar to that among Latinos receiving HIV care in the USA 5, 6, 7. Regarding prevention, mother‐to‐child transmission (MTCT) has substantially decreased as provision of ART has ramped up 8.

And in the past decade, concerted efforts by activists, advocates, committed politicians, scientists and many others, have slowly advanced the recognition of rights of minorities 9, 10. For example, there has been a substantial increase during this century in the number of countries where same‐sex sexual activity has been de‐penalized, equal rights of marriage and child adoption extended to same‐sex couples, and strong legal protections against discrimination and violence based on sexual orientation or gender identity implemented 11. Nevertheless, these advances are threatened by the recent political and economic backlash throughout the entire American continent, adding to the challenges lying ahead in controlling the HIV epidemic 12, 13.

First and foremost, discrimination and violence against sexual minorities, in particular, transgender people, is far from over: Seventy‐eight percent of transgender women reported to have been murdered from 2008 to 2015 worldwide were killed in Latin America 14. Such levels of stigma and discrimination remain significant barriers to protection of even the most basic human rights.

Similarly, the estimated 2 million people who inject drugs living in Latin America have been neglected despite being one of the groups with the highest HIV prevalence (7.4%) 15. As a result, there is an almost complete absence of reliable data on access to HIV and harm reduction services, which has made it difficult to design, fund and implement evidence‐based strategies to reduce HIV incidence among people who inject drugs in the region 16.

The impact of major trade routes, dynamics of regional consumption and the heterogeneity of constantly changing drug enforcement policies and activities on risk behaviours makes this population a moving target 17. However, punitive laws and practices and the sheer lack of political remain major barriers for drug users to access HIV prevention and treatment services 18. As long as there is a broad social and political consensus that continues to frame drug use and dependence as a criminal law concern, rather than as public health and/or human rights issues, costly, wasteful and ineffective punitive interventions will continue 19.

During the 38 years of the HIV/AIDS epidemic, we have learned that it can be fuelled in environments where human rights for vulnerable populations are limited. We have also learned that coercive laws and misguided policies aiming to ban sex work and drug use may actually promote HIV transmission 20.

There is no straightforward solution since policies to reduce stigma and discrimination, prevent violence and improve access to harm reduction services must be supported and implemented by the same governments that are currently undermining the already adverse social and political environment in some countries. Organized efforts by international and local civil society organizations supported by progressive governments, intergovernmental agencies and academia might lead to the launch of political pressure initiatives to resist and contain the current adverse political trends.

Although the continuum of care in Latin American countries has improved over time 21, 22, none have reached the 90‐90‐90 targets established by UNAIDS (Figure 1). The annual numbers of new infections have barely changed in the past two decades, overall mortality reductions have been heterogeneous and lower than expected 6, and AIDS‐related conditions continue to be the leading causes of death among PLHIV in the region despite achievements in access to ART 1. This might be explained by the persistently high frequency of late HIV diagnosis, which still occurs in almost half of diagnosed adults in Latin America 22, 23.

**Figure 1:**
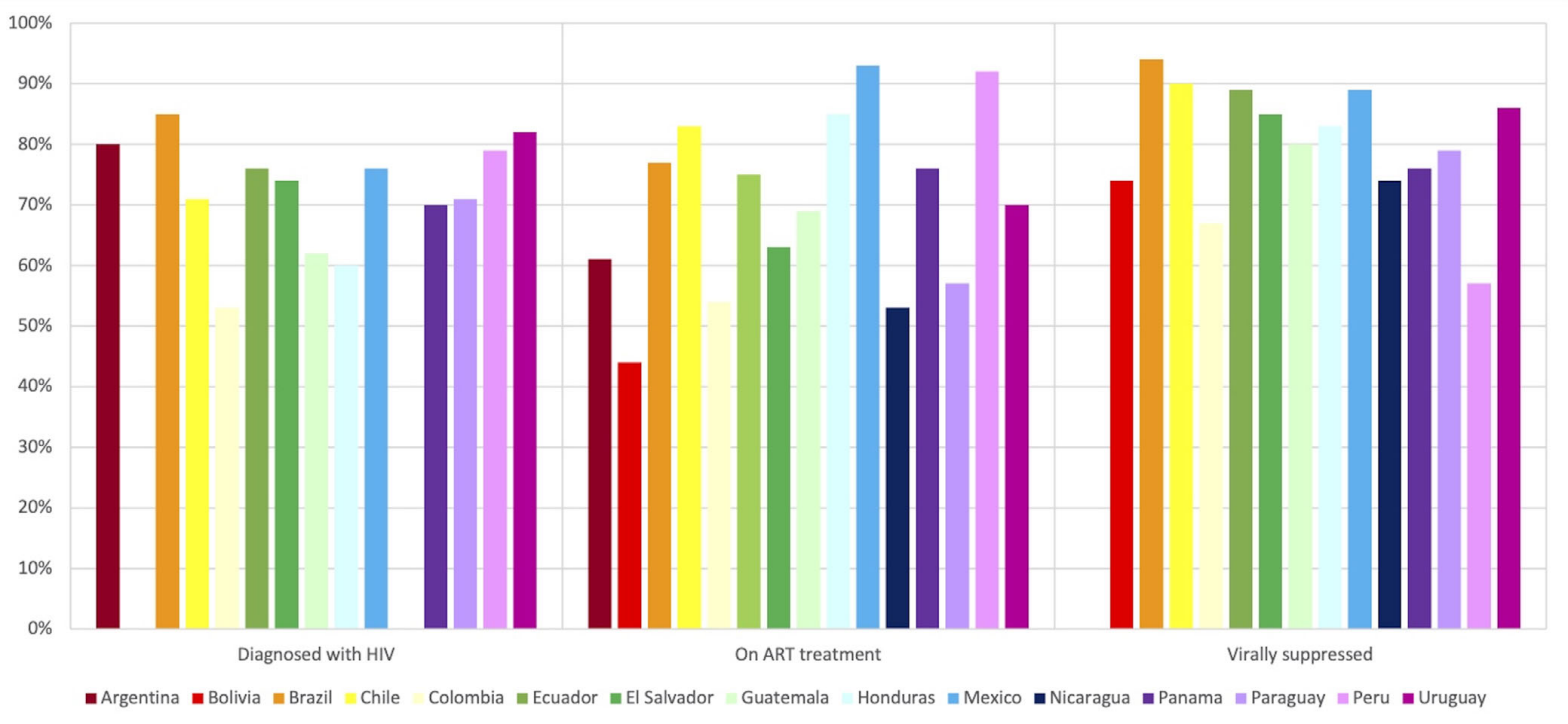
The current status of meeting the 90‐90‐90 targets in Latin America. Sources: UNAIDS data 2019. Bulletin on HIV, AIDS and STis in Argentina, December 2018. Joint United Nations Program for AIDS‐UNAIDS, Institute of Public Health. Registration of the care centers of the Public Assistance Network; Superintendence of Health. Current HIV‐AIDS in Peru. General Di rection of Medicines, Supplies and Drugs (DIGEMID).

If we aim to fully achieve the 90‐90‐90 targets to control the HIV epidemic and end it as a public health problem, much more must be done to rapidly reduce the proportion of people unaware of their HIV status. Innovative strategies and tools to increase access to HIV screening tests are urgently needed. Strategies to demedicalize HIV counselling and testing services to make them accessible for hard‐to‐reach vulnerable groups and the implementation of proven self‐testing models will be essential to achieve the target of having 90% of PLHIV being aware of their status – the first 90. This may require legislative and administrative changes in many Latin American countries in addition to increasing funds to allow for scale‐up of testing strategies, immediate linkage and same‐day ART initiation programmes with simplified, integrase inhibitor‐based regimens 24, 25, 26.

In terms of prevention, efforts to eliminate MTCT have clearly been insufficient (with the exemption of Cuba and six of the English‐speaking Caribbean nations and territories) 8, 18. Broader improvements in healthcare systems are needed; these include strengthening of prenatal/maternal care services paired with improved access to HIV testing for all pregnant women and coordination with ART programmes to immediately initiate them on ART 9. Countries should build on the experiences in Cuba and the Caribbean to eliminate MTCT.

Furthermore, pre‐exposure prophylaxis (PrEP) is unacceptably scarce across the region. Programmes must be rapidly expanded as PrEP has been shown to control HIV transmission in concentrated epidemics elsewhere 27. A multinational implementation project (ImPrEP), funded by Unitaid, national governments and other partners, is ongoing and is providing PrEP services to 7500 vulnerable gay men and transgender women in Brazil, Mexico and Peru with encouraging results, such as high retention and adherence (above 80% and 90% respectively) 28, 29. Nevertheless, successful implementation of large‐scale PrEP programmes urgently needs political commitment, leadership, civil society advocates and the involvement of scientific and academic communities to move them forward.

Finally, the consequences of migration for the HIV epidemic in our region have rarely been considered in relation to the implementation of HIV prevention, treatment and care programmes. In addition to long‐established migration patterns 30, recent political conflicts and economic instability in Central America and Venezuela have fostered massive waves of immigration throughout the region. UNAIDS estimated in 2017, only 49% of the 120,000 PLHIV in Venezuela had access to ART and <7% were virally suppressed 31. Alarmingly, none of the blood banks in the country are reported to have supplies to test for HIV. Difficulties in ART acquisition in Venezuela in 2017 culminated in widespread drug shortages in 2018 32. As a result, nearly nine of the 10 PLHIV in Venezuela stopped receiving ART and some of them migrated to other countries in search of treatment 31. This could present one of the more dramatic examples on how migration significantly impacts HIV care and control programmes; however, beyond the ongoing crisis, Latin America has historically been the origin, destination and transit of regional migrants.

Around 40 million Latin Americans live outside their native countries. Up to half of this population might have migrated within the region, and migrants constantly cross boundaries within the region 33, 34, 35. Large‐scale migration across borders increases vulnerability to HIV/AIDS and other sexually transmitted infections, probably through mechanisms that include sexual exploitation during human trafficking, exposure to sexual violence and new sexual partners, different social and sexual norms and different HIV prevalence rates 36. Moreover the continuity of ART provision for individuals is threatened during migration. Innovative solutions to this situation go beyond any single nation and will require a coordinated plan to ensure that people migrating across borders have their fundamental human right to health guaranteed. Health systems in countries receiving migrants from Venezuela and elsewhere, especially those of Argentina, Chile, Colombia, Mexico and Peru, should be strengthened so that healthcare needs of migrants and refugees can be met without negative consequences for local HIV programmes 37.

A regional leadership and collaboration, internationally funded, could assist and support migrants and provide or coordinate services, including healthcare, across borders, building on the previous experiences and leadership of UNICEF and the UN International Organization for Migration.

In the past, the diversity of the HIV epidemic in Latin America has been tackled by progressive and often innovative treatment and prevention approaches, together with intense community engagement. This has succeed in spite of the challenges posed by conservative groups and governments, migration, natural disasters and civil unrest. However, our current situation has changed, with new governments, either ideologically motivated or in response to economic crises, reducing public health budgets and seeking to suppress hard‐won liberties and rights.

While we can look back at how much we have accomplished and learn from our history and from each other, our epidemic demands a renewed effort, one that integrates new tools at our disposal and follows a clear path built by science, with the strength of will of those who lived with HIV and AIDS in the past.

## Competing interests

All authors confirm that they have no conflicts of interest.

## Authors' contributions

BCR, PFBZ and CC developed the conceptualization and design of the viewpoint and wrote the manuscript. OS, AP and BG contributed to conception, design and reviewing of the manuscript. MM, JSM and PC contributed to design and reviewing of the manuscript. All authors have read and approved the final manuscript.
